# P-glycoprotein Dysfunction Contributes to Hepatic Steatosis and Obesity in Mice

**DOI:** 10.1371/journal.pone.0023614

**Published:** 2011-09-16

**Authors:** Magali Foucaud-Vignault, Zeina Soayfane, Cécile Ménez, Justine Bertrand-Michel, Pascal Guy Pierre Martin, Hervé Guillou, Xavier Collet, Anne Lespine

**Affiliations:** 1 UMR1331, INP, UPS, TOXALIM, INRA, Toulouse, France; 2 UMR 1048I, NSERM, Toulouse, France; 3 Institut de Maladies Métaboliques et Cardiovasculaires, UPS/INSERM, Toulouse, France; 4 IFR150, Toulouse, France; University of Illinois at Chicago, United States of America

## Abstract

Although the main role of P-glycoprotein (Pgp) is to extrude a broad range of xenochemicals and to protect the organism against xenotoxicity, it also transports a large range of endogenous lipids. Using mice lacking Pgp, we have investigated the possible involvement of Pgp in lipid homeostasis *in vivo*. In a long term study, we have followed the food intake, body status and lipid markers in plasma and liver of wild-type and mdr1ab^-/-^ mice over 35 weeks. Pgp-deficient mice showed excess weight, hypertrophy of adipose mass, high insulin and glucose levels in plasma. Some of these metabolic disruptions appeared earlier in Pgp-deficient mice fed high-fat diet. Moreover, hepatosteatosis with increased expression of genes involved in liver detoxification and in *de novo* lipid synthesis occurred in Pgp-deficient mice. Overall, Pgp deficiency clearly induced obesity in FVB genetic background, which is known to be resistant to diet-induced obesity. These data reinforce the finding that Pgp gene could be a contributing factor and possibly a relevant marker for lipid disorder and obesity. Subsequent to Pgp deficiency, changes in body availabilities of lipids or any Pgp substrates may affect metabolic pathways that favour the occurrence of obesity. This is of special concern because people are often facing simultaneous exposition to many xenochemicals, which inhibits Pgp, and an excess in lipid dietary intake that may contribute to the high prevalence of obesity in our occidental societies.

## Introduction

The P-glycoprotein (Pgp, ABCB1) is a membrane efflux transporter that belongs to the ATP-binding cassette family, which in humans is the product of the *ABCB1* gene. P-glycoprotein was first identified as a factor involved in multidrug resistance in mammalian tumor cells [1] and was subsequently found to be physiologically expressed at the apical surface of epithelial cells [2]. The main role of Pgp is to actively pump xenobiotics out of cells and out of the organism. Its widespread tissue distribution and large substrate specificity confer to this protein a strategic role in the protection of the tissues and of the whole body against xenobiotic toxicity [3]. For example, Pgp is located in the intestine and provides a solid protection by extruding many food contaminants or naturally occurring dietary substrates back into the lumen. Similarly, Pgp on the blood-brain-barrier effluxes compounds out of the brain thereby limiting their neurotoxicity. By extension, it also limits the intestinal absorption and brain penetration of drugs which are Pgp substrates, thus limiting their efficiency. Using Pgp-deficient mice, the pivotal role of Pgp in the pharmaco- and toxicokinetics of many substrate drugs such as digoxin, dexamethasone, cyclosporine or ivermectin has been demonstrated [4]-[7].

Besides its clear role in the transport of pharmaceuticals and contaminants, Pgp is also involved in the movement of endogenous molecules such as cholesterol, phospholipids and sphingolipids [8] and a variety of steroids [9]. Pgp participates in the cellular uptake of exogenous cholesterol in recombinant cells overexpressing Pgp [10]. Pgp is predominantly localised in lipid rafts which are cholesterol-rich membrane microdomains and it is involved in cholesterol transbilayer transfer through membranes. In addition, cholesterol content of membranes affects Pgp transport activity by modulating the ATPase activity [11]-[13]. *In vivo,* after cholesterol oral loading in Pgp-deficient mice there was no change in cholesterol intestinal absorption but an increase of cholesterol ester concentration in the liver [14]. However, until now, no clear phenotype has been reported for Pgp-deficient mice in the absence of drug challenge [5] ruling out any substantial contribution of Pgp in lipid turnover *in vivo*. But it is noteworthy that the FVB genetic background of the available Pgp-deficient mice is known to be resistant to diet-induced lipid disorders which certainly make elusive any change in lipid homeostasis [15].

Indeed, studies in human populations are in favour of a link between cholesterol levels and *ABCB1* gene polymorphisms [16]-[18]. Most interestingly, a study performed in a Japanese cohort revealed that a single nucleotide polymorphism on *ABCB1* gene was associated with obesity [19]. In addition to all these data and in favour of some involvement of Pgp in lipid homeostasis, during the course of studies performed in our laboratory, we observed an intriguing accumulation of abdominal fat in the Pgp-deficient animals.

Given the possibility that Pgp is involved in lipid trafficking and because accumulation of abdominal fat can foreshadow a propensity to obesity, the aim of our work was to study the impact of Pgp deficiency on lipid homeostasis in the whole organism. Therefore, we have performed a long term study and followed body weight change and lipid parameters in Pgp-deficient (mdr1ab^-/-^) and wild-type mice**.** We have then measured the expression of pivotal genes involved in lipid metabolism (*acox, cd36, fasn, scd-1)* and xenobiotic detoxification (*cyp2b10* and *cyp3a11*) which can be regulated either by lipids or by xenobiotics, respectively. For the first time, we have demonstrated that the lack of Pgp is associated with overweight and liver steatosis in mice arguing in favour of a role for Pgp in maintaining lipid homeostasis and preventing obesity.

## Materials and Methods

### Ethics Statement

*In vivo* studies were conducted in mice under European laws on the protection of animals (86/609/EEC). Protocols are performed under procedure and principal for good clinical practice (CVMP/VICH 59598). The protocols for experimentation on rodents used in this manuscript have been approved by the local institutional animal care and ethics committee which is the “Direction Départementale des Services Vétérinaires de Haute-Garonne”. The specific approval number for this study approval is B31555-25.

### Animal housing

Wild-type and the Pgp knock-out mdr1ab^-/-^ mice with a FVB genetic background were obtained from Taconic (NY, USA). In rodents, there are two Pgps encoded by *abc1a* and *abc1b* genes and mdr1ab^-/-^ mice were deficient for the two gene products [5], [6]. Mice were housed at INRA's transgenic rodent facility at 22±2°C under 12-hour light/dark cycles. Animals sampling was designed to reduce the influence of interfering parameters such as litter specificity (seven to nine different litters for a ten animals group). Mice received a standard chow diet recommended for the breeding and rearing of rodents (Harlan Teklad TRM Rat/Mouse Diet; Harlan Teklad, Gannat, France). Water and food were available *ad libitum*.

### Experimental design

Mice of both genotypes (10–12 males and 10 females per group) were weighted and the food intake was measured weekly from weaning to 35 weeks of age. At 25 weeks, food was withdrawn 2 hr prior to euthanasia and 5 animals of each group were anesthetized by intraperitoneal administration of xylazine and ketamine cocktail (53 and 10 mg/kg bw, respectively). Blood was collected from the orbital sinus vein. Several white adipose pads (subcutaneous inguinal, perigonadal, perirenal, mesenteric) and liver were then excised and weighted. At 35 weeks, the rest of the animals (n = 5-6 per groups) were processed as described above for blood and tissue sampling. Plasma and liver were frozen in liquid nitrogen and stored at −80°C until analysis.

### High-fat diet study

Mice were assigned to normal diet (wild-type *n* = 6 and mdr1ab^-/-^ *n* = 6) and high-fat diet HFD (wild-type *n* = 6 and mdr1ab^-/-^ *n* = 6) (DIO Research diet, Jackson Laboratory, Brogaarden, Denmark) for 12 and 25 weeks. Energy contents of the specific diets were (% kcals): 20% protein, 70% carbohydrate, and 10% fat for normal diet; 20% protein, 35% carbohydrate, and 45% fat for HFD. Animals were weighted and food intake was measured all along the experiment. Three animals of each group were sacrificed at 15 and at 25 weeks. Blood was withdrawn and plasma and liver were collected and frozen for further analysis.

### Plasma analysis

Plasma insulin was quantified by using a kit ELISA for mouse insulin (EUROBIO, France). Plasma glucose was determined by the glucose oxidase method. The following parameters: alkaline phosphatase (ALP), alanine transaminase (ALT), aspartate transaminase (AST), lactate dehydrogenase (LDH), lipase, HDL cholesterol (HDLc) LDL cholesterol (LDLc), triglycerides and free fatty acids, were measured using a COBAS-MIRA+ analyser (Service Phénotypage, IFR150/BMT, France).

### Lipid analysis in the liver

Lipids were extracted from the liver according to Bligh and Dyer [20]. Briefly, liver tissue was homogenized in 2 ml of water and 5 ml of chloroform-methanol (1∶1, v/v). Samples were vortexed and centrifuged at 1500 rpm for 2 minutes at 4°C. The lower organic phase was removed, dried under nitrogen gas, taken up in 160 µl ethyl acetate and dried again under nitrogen gas. The dried lipid samples were dissolved again in 20 µl ethyl acetate. Triglycerides, total cholesterol and cholesterol esters were analyzed and quantified by gas chromatography [21] (Plateau de Lipidomique, IFR150/BMT, Toulouse).

### Oil red O staining and histological analysis

A part of the liver was embedding in OCT in Tissue-Teck (Miles, inc., Kankakee, IL), then the entire block was frozen in isopentane prior to storage at −80°C. The histological analyses were performed on serial sections (6 µm thick), fixed in 10% buffered formalin, stained with oil red O and counterstained with hematoxylin (original magnification X200, Plateau d'Histopathologie Expérimentale, IFR150/BMT, Toulouse).

### Liver mRNA abundance

For RNA extraction, a part of the large lobe of the liver was collected and immediately frozen in liquid nitrogen before storage at −80°C. Total RNA was extracted with TRIzol reagent (Invitrogen, France) from liver tissues. For real-time quantitative PCR (Q-PCR), total RNA samples (2 µg) were reverse-transcribed with SuperScript II (Invitrogen). All assays were performed on an ABI Prism 7000 (Applied Biosystems, Courtaboeuf, France) using standard PCR conditions. Primers and Taqman probes for TATA-box binding protein *tbp* (NM_013684), *acox* (NM_015729), *cd36* (NM_007643), *fasn* (NM_007988), *scd-1* (NM_009127), *pltp* (NM_011125), *cyp7a1* (NM_007825), *cyp2b10* (NM_009999), *cyp3a11* (NM_007818) were purchased from Applied Biosystems Assays-on-Demand. Primers for SYBR Green assays were as follows: *tbp*-F, 5′-ACTTCGTGCAAGAAATGCTGAA-3′; *tbp*-R, 5′-GCAGTTGTCC-GTGGCTCTCT-3′; *acox*-F, 5′-CAGACCCTGAAGAAATCATGTGG-3′; *acox*-R, 5′-CAGGAACATGCCCAA-GTGAAG-3′; *cd36*-F, 5′- GTTAAACAAAGAGGTCCTTACACATACAG-3′; *cd36*-R, 5′- CAGTGAAGGCTCAAAGATGGC-3′; *fasn*-F, 5′-AGTCAGCTATGAAGCAATTGTGGA-3′; *fasn*-R, 5′-CACCCAGACGCCAGTGTTC-3′; *scd-1*-F, 5′-.CCGGAGACCCCTTAGATCGA-3′; *scd-1*-R, 5′- TAGCCTGTAAAAGATTTCTGCAAACC-3′; *pltp-F, 5* ′*-* GGATTAAAGTGTCCAATGTCTCCTG-3′; *pltp-R, 5* ′*-* GTGGAGAAAAAGTTATACATCCTCCTG-3′; *cyp7a1-F, 5* ′*-* ACATGGTGACACTTTCACTGTCTTC-3′; *cyp7a1-R, 5* ′*-* GAACTTCTGAAAGCTTAATTGTTTTGG-3′; *cyp2b10*-F, 5′-TTTCTGCCCTTCTCAACAGGAA-3′; *cyp2b10*-R, 5′-ATGGACGTGAAGAAAAGGAAC-AAC-3′; *cyp3a11*-F, 5′-.TCACACACACAGTTGTAGGCAG-AA-3′; *cyp3a11*-R, 5′-GTTTACGAGTCCCATATCGGTAGAG-3′. A pool of all complementary DNA samples was used to generate calibration curves. All Q-PCR data were normalized by TATA-box binding protein mRNA levels.

### Statistical analyses

The experimental values are expressed as mean ± standard error of the mean (s.e.m). Statistical analyses were performed using a three-way factorial ANOVA model. A three-way factorial ANOVA model with repeated measures was used in the case where more than one observation came from the same animal. Multiple comparisons of means were performed with Tukey test. Statistical significance was accepted as *p<*0.05.

## Results

### Body weight and food intake in wild-type versus Pgp-deficient mice

**Figure 1:**
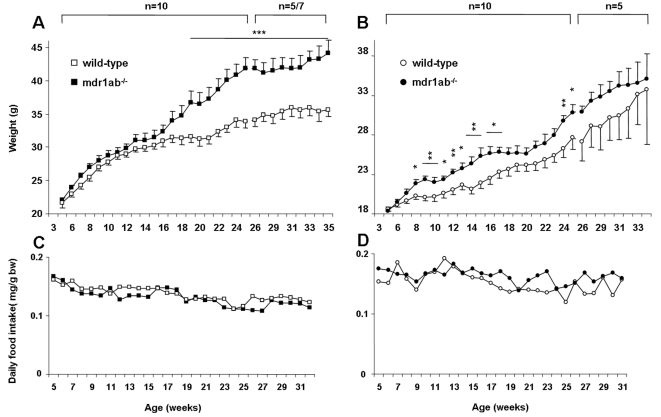
Time kinetic of body weight and food intake in wild-type versus mdr1ab^-/-^ mice. The weights were recorded weekly from weaning to 35-weeks of age for male (Fig. 1A) or female (Fig. 1B) wild-type (open symbols) and mdr1ab^-/-^ (close symbols) mice fed a standard chow diet. Results are means ± s.e.m of 5 to 10 animals. Differences are considered significant when *, *p*<0.05; **, *p*<0.01; ***, *p*<0.001. Daily food intakes of males (Fig. 1C) and females (Fig. 1D) were calculated weekly by monitoring the total food consumption for wild-type (open symbols) and mdr1ab^-/-^ (close symbols) mice, measured as mean food consumption for two to three mice caged together. Results are expressed per gram of body weight and are the means of 3 values per subgroup.

Mice were fed with a standard chow diet throughout the study and weekly recording of body weight (bw) was performed over 35 weeks. No phenotypic abnormality was observed in young Pgp-deficient mice for up to 7 weeks of age for female, and 15 weeks of age for male mice, and they were morphologically undistinguishable from age-matched wild-type mice (Fig. 1A and 1B). After these respective ages, growth curves showed a significant increase in body weight for females (*p<*0.05) and males (*p<*0.001) mdr1ab^-/-^ in comparison with wild-type mice. The weight difference appeared earlier in females, as early as 8 weeks of age, than in males (18 weeks) but the difference between wild-type and Pgp-deficient mice was greater in males. Daily food intake was monitored throughout the experiment and no difference in calorie intake was observed in mdr1ab^-/-^ males or females, compared with wild-type (Fig. 1C and 1D). Given that in our experiment the weight differences were more pronounced in male mice when compared with females, the subsequent analyses were performed in males.

### Influence of Pgp deficiency on tissue weight in mice

**Figure 2:**
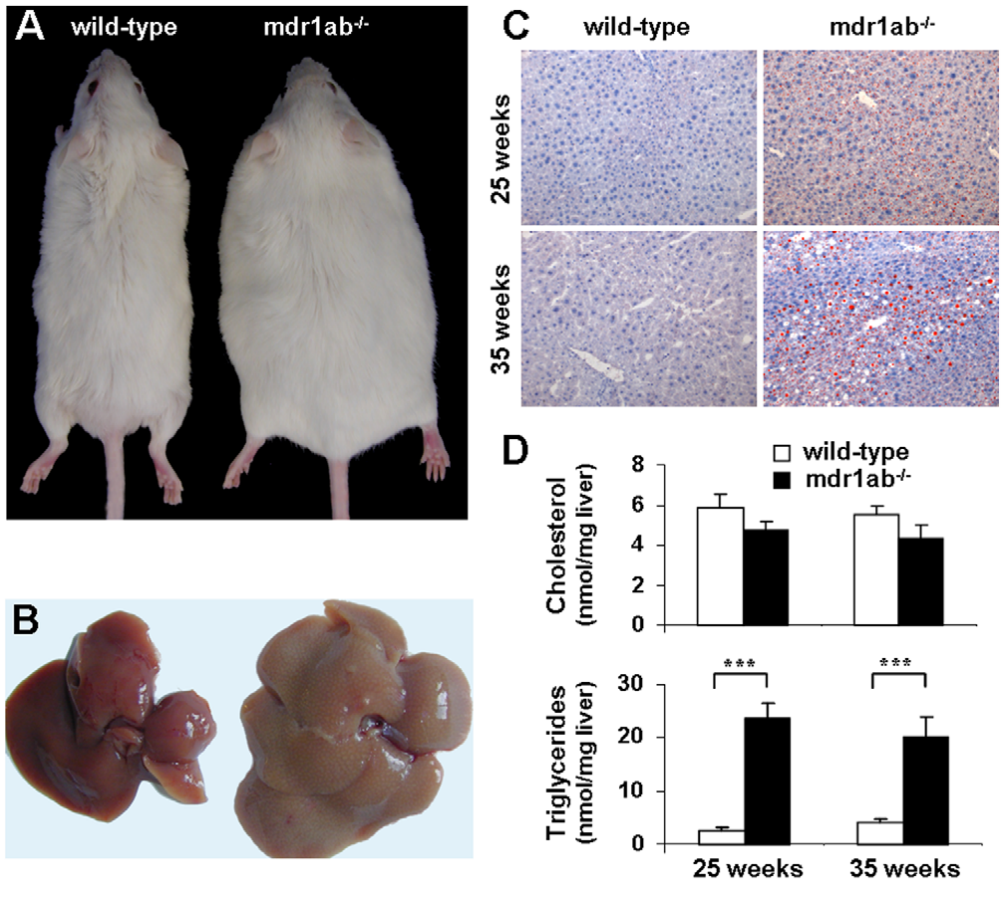
Morphological comparison and liver lipid content in wild type and mdr1ab^-/-^ males. 2A. Whole body. 2B. Respective livers at 35 weeks of age. 2C. Oil red O staining of liver sections. Livers were collected from 25- or 35-week old mice and kept frozen in OCT at −80°C until analysis. 2D. Lipid content of liver. Total cholesterol (upper histogram) and triglycerides (lower histogram) in liquid-frozen liver samples from 25- and 35- week old mice, were quantified by liquid chromatography in wild-type (open bars) and mdr1ab^-/-^ (solid bars) males. Results are expressed as means ± s.e.m of 5 to 7 mice. Differences are considered significant when *p<*0.05. ***, *p*<0.001.

To get insights into the origin of the overweight phenotype in Pgp-deficient male mice, liver and white adipose tissue from four anatomical sites (subcutaneous inguinal, periepididymal, perirenal and mesenteric) were weighed and their relative contribution to the body weight were calculated in 25- and 35-week old mice (Table 1). Liver weight was increased in Pgp deficiency to a similar extent as whole body weight (Fig. 2A and 2B). In the meantime, the total mass of white adipose tissues was strongly increased at the two periods in Pgp-deficient mice (Table 1). At 25 weeks of age, the contribution of the subcutaneous inguinal, perirenal, mesenteric and periepidydimal fat pads to the bw of mdr1ab^-/-^ male mice increased by 2.6-, 2.3-, 2.7- and 1.2-fold, respectively, in Pgp-deficient mice compared with wild-type. At 35 weeks of age, the mass of all the white fat pads were increased in Pgp deficiency but the differences in their relative contribution to the body weight between wild-type and Pgp-deficient mice were less pronounced than earlier, certainly related to the increase in mass of adipose tissues which was observed with age.

**Table 1:** Liver and adipose tissue contributions to the body weight in wild-type versus mdr1ab^-/-^ mice.

	25 weeks			35 weeks		
	Wild-type (n = 6)	Mdr1ab^-/-^ (n = 5)	F	Wild-type (n = 6)	Mdr1ab^-/-^ (n = 5)	F
**Weight** (g)	31.9±0.6	43.9±1.0^a^	1.38	36.2±1.3	44.1±2.0^b^	1.22
**Liver** (% of bw)	4.94±0.28	4.87±0.21	0.98	4.63±0.16	4.88±0.21	1.05
**White adipose tissue** (% of bw)						
*subcutaneous inguinal*	1.05±0.41	2.72±0.13^b^	2.58	1.35±0.20	4.05±0.50^a^	2.99
*perigonadal*	1.86±0.31	2.18±0.22	1.17	2.37±0.27	3.55±0.17^b^	1.50
*perirenal*	0.42±0.09	0.98±0.02^b^	2.32	0.91±0.18	1.29±0.10	1.42
*mesenteric*	0.75±0.06	2.02±0.22^a^	2.69	1.88±0.19	2.26±0.15	1.21
*Total*	4.09±0.65	7.91±0.53^b^	1.94	6.51±0.69	11.15±0.46^a^	1.71

Mice were sacrificed at 25 and 35 weeks of age and discrete fat pads from five anatomical sites were dissected and weighed. WAT refers to white adipose tissue. Total WAT weight represents the sum of the data reported for the four WAT sites considered in the study. Body weight is expressed in gram and tissue weights are expressed as percentage of body weight. F is the ratio of mdr1ab^-/-^ versus wild-type. Results are means ± s.e.m. Statistical relevance of data is assessed by *p* values.

a *p*<0.05;

b *p*<0.01.

### Analysis of enzymes, lipids and glucose in plasma

Biological parameters were measured in mice plasma and were reported in Table 2. At 25 weeks, none of the plasma parameters explored were different between Pgp-deficient compared with wild-type mice, apart from insulin levels, which were strongly increased (0.58±0.39 and 6.62±2.11 ng/mL, *p<*0.05). At 35 weeks, the alkaline phosphatase (ALP) and alanine transaminase (ALT) activities were increased in Pgp-deficient mice (*p<*0.01 and ns, respectively, compared with wild-type). In the mean time, aspartate transaminase (AST) activity was significantly decreased (1.5 fold, *p<*0.05) and lipase and lactate dehydrogenase (LDH) activities were unchanged. Total cholesterol concentration in plasma was slightly but significantly higher in mdr1ab^-/-^ mice compared with wild-type (4.03±0.13 versus 3.46±0.17 mmol/L, *p<*0.05). At the same time, HDL cholesterol was significantly enhanced in mdr1ab^-/-^ mice whereas no change was observed for plasma LDL cholesterol level. The triglycerides tended to be decreased in the plasma of Pgp-deficient mice while the free fatty acid levels were unchanged. Overall, in mdr1ab^-/-^ mice, plasma glucose and insulin concentrations were increased compared with wild-type (1.5- and 23-fold, respectively, *p<*0.05).

**Table 2:** Biomarker analysis in plasma of wild-type and mdr1ab^-/-^ mice.

	25 weeks			35 weeks		
	Wild-type	Mdr1ab^-/-^	F	Wild-type	Mdr1ab^-/-^	F
ALP (U/L)	18.3±10.3	16.0±5.0	0.87	4.0±1.5	14.0±1.2 b	3.50
ALT (U/L)	30.7±7.4	101.3±33.1	3.30	76.6±23.6	134.8±32.1	1.76
AST (U/L)	107.7±27.2	199.0±39.6	1.85	530.8±70.7	277.6±42.7 a	0.52
LDH (U/L)	984±268	2074±558	2.11	4764±596	3869±641	0.81
Lipase (U/L)	21.0±1.2	26.3±2.4	1.25	29.0±2.0	37.6±10.2	1.30
Cholesterol (mmol/L)	3.26±0.11	3.23±0.01	0.99	3.46±0.17	4.03±0.13 a	1.16
HDLc (mmol/L)	3.05±0.09	2.73±0.14	0.89	3.11±0.18	3.67±0.13 a	1.18
LDLc (mmol/L)	0.10±0.01	0.10±0.01	1.00	0.16±0.02	0.16±0.02	1.00
Triglycerides (mmol/L)	0.86±0.07	0.92±0.04	1.07	2.13±0.30	1.46±0.30	0.69
Free Fatty Acid (mmol/L)	2.88±0.12	3.00±0.15	1.04	1.10±0.05	1.12±0.06	1.02
Glucose (mmol/L)	18.3±1.7	16.1±1.3	0.88	13.4±1.1	20.9±3.0 a	1.56
Insulin (ng/mL)	0.58±0.39	6.62±2.11^a^	11.4	0.17±0.02	4.73±1.82 a	27.8

Individual plasma samples collected at 25 and 35 weeks were analyzed. Results are expressed as means ± S.E.M. of 5 analysis for wild-type and mdr1ab^-/-^ mice, except for ALP analysis where only 3 samples of each group were analysed. F is the ratio of mdr1ab^-/-^ versus wild-type.

a *p*<0.05;

b *p*<0.01.

ALP: Alkaline phosphatase; ALT: Alanine transaminase; AST: Aspartate transaminase; LDH: Lactate dehydrogenase; HDLc: High density lipoprotein cholesterol; LDLc: Low density lipoprotein cholesterol.

### Liver morphology and composition

**Figure 3:**
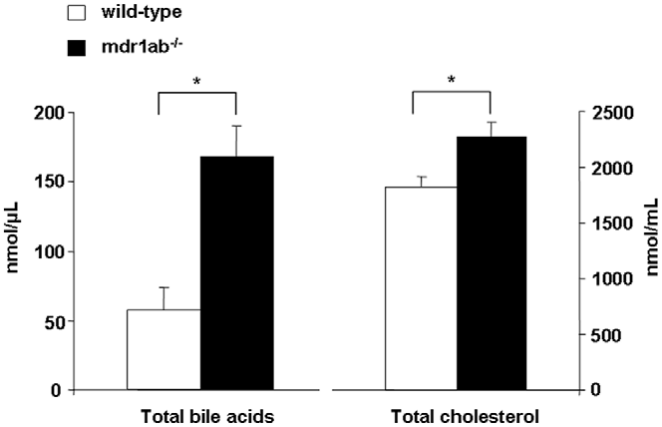
Lipid content in bile of wild-type and mdr1ab^-/-^ mice. Cholesterol and bile acids were quantified in bile in wild-type (open bars) and mdr1ab^-/-^ (solid bars) 35-week old male mice. Results are expressed as means ± s.e.m of 5 mice. Differences are considered significant when *, *p*<0.05; **, *p*<0.01; ***, *p*<0.001.

Major hepatic abnormalities were observed at necropsy of the Pgp-deficient liver at 35 weeks, with a pale steatotic colour of the liver and a regular pattern of pale dots displayed on all lobe surfaces (Fig. 2B). These abnormalities, which are strongly evocative of hepatic steatosis, were also observed in some of the 25-week old Pgp-deficient livers (1 over 3). Histological analyses of neutral lipid in oil red O stained sections of frozen livers, revealed at 25 and 35 weeks clear accumulation of intracellular fat droplets restricted to the centrilobular area of mdr1ab^-/-^ livers (Fig. 2C). At 25-weeks, most of the fat droplets appear bigger when compare to liver at 35 weeks where fat droplets were smaller. Normal hepatocytes were restricted to periportal zones while wide centrilobular areas suggested fat storage. At 25 weeks, hepatic triglyceride concentrations were 10-fold greater in mdr1ab^-/-^ livers than in wild-type and this difference was maintained at 35 weeks (Fig. 2D, 20.2±3.7 and 4.3±0.6 nmol/mg, respectively, *p<*0.001). In addition, in Pgp deficient mice at both ages, the total cholesterol content in the liver was unchanged (Fig 2D, ns) while cholesterol ester concentration tended to be higher (2-fold, ns, not shown). In addition, the total bile acids and cholesterol concentrations were significantly higher in Pgp-deficient mice compared with wild-type (Fig. 3).

### Influence of high fat diet on Pgp-deficient mice

**Figure 4:**
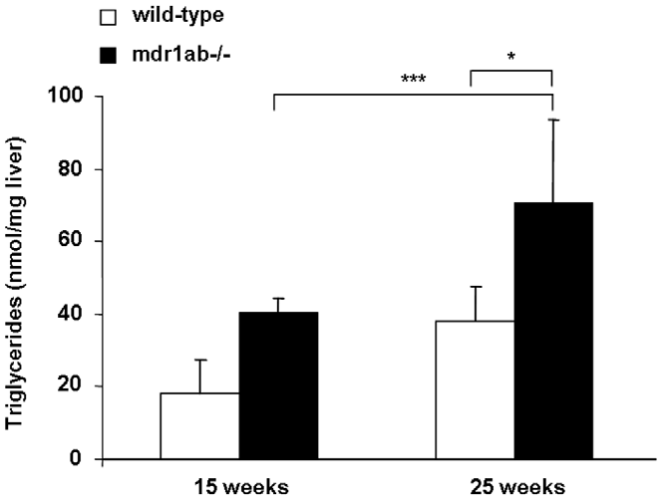
Influence of high fat diet (HFD) on hepatic triglyceride concentrations in wild-type and mdr1ab^-/-^ mice. Mice were fed with HFD during 25 weeks. Triglycerides have been quantified in liver samples from 15- or 25-week old wild-type (open bars) and mdr1ab^-/-^ (solid bars) mice. Results are expressed as nmol per mg of liver and are means ± s.e.m of 3 values at 15-week old and 5 at 25-week old. Differences are considered significant when *, *p*<0.05; **, *p*<0.01.

In order to test the impact of a high fat diet, a new study was conducted with mdr1ab^-/-^ and wild-type mice fed a high fat-diet (HFD) from weaning to 25 weeks. No difference in body weight between the two different groups fed HFD was observed during the experiment suggesting that the overweight observed in Pgp-deficient mice fed normal chow diet was maximal. By contrast, wild-type mice fed HFD gained weight compared to those fed normal chow diet. At 25 weeks, plasma glucose concentrations were increased in both groups fed HFD when compared to mice fed normal diet and the increase was greater in the Pgp-deficient mice (2.1-fold, *p<*0.01) than in wild-type mice (1.5-fold, *p<*0.05). In the mean time, the triglycerides level in plasma were unchanged by HFD in wild-type mice and were increased in Pgp-deficient mice fed HFD (Table 3, *p<*0.05). In Pgp-deficient mice fed HFD, the liver showed apparent abnormalities as soon as 15 weeks and at 25 weeks of age. In age-matched animals fed a normal diet, no liver abnormality was observed in wild-type mice at 25 weeks while some of Pgp-deficient animals displayed apparent liver abnormality. Lipids measurements performed in the liver showed that at 15-week old, Pgp-deficient mice fed HFD tend to have higher liver triglyceride concentration compared with wild-type mice (30.1±6.1 and 18.2±6.1 nmol/mg, respectively, ns). At 25 weeks of age, triglyceride concentration in the liver was higher in both strain fed HFD compared with 15-week old mice, but the increase was more pronounced in the Pgp-deficient mice than in wild-type (70.6±18.8 and 31.5±3.7 nmol/mg tissue, respectively, *p<*0.01, Fig. 4). In the mean time, a 2-fold increase in cholesterol ester concentration was measured in the liver of Pgp-deficient mice compared with wild-type (4.42±0.4 and 8.75±1.04 ng/mg respectively, *p<*0.05, not shown).

**Table 3:** Influence of high fat diet on plasma glucose and triglyceride concentrations in wild-type and mdr1ab^-/-^ mice at 25 weeks of age.

		Wild-type	Mdr1ab^-/-^
**Glucose (mmol/L)**	*Standard diet*	18.3±1.7	16.1±1.3
	*HFD*	26.9±1.7 *	34.3±1.3 a **
**Triglyceride (mmol/L)**	*Standard diet*	0.80±0.07	0.92±0.04
	*HFD*	0.70±0.03	0.99±0.05 a

Mice were fed from weaning to 25 weeks of age whether with a standard chow diet or a high fat diet (HFD). Glucose and triglycerides were analysed in plasma samples from wild-type and mdr1ab^-/-^ mice. Results are expressed as means ± s.e.m. of 3 to 5 animals.

a *p*<0.05 when compared with wild-type.

*p<0.05;

**p<0.05, when compared with standard diet.

### Analysis of xenobiotics and lipids target genes

**Figure 5:**
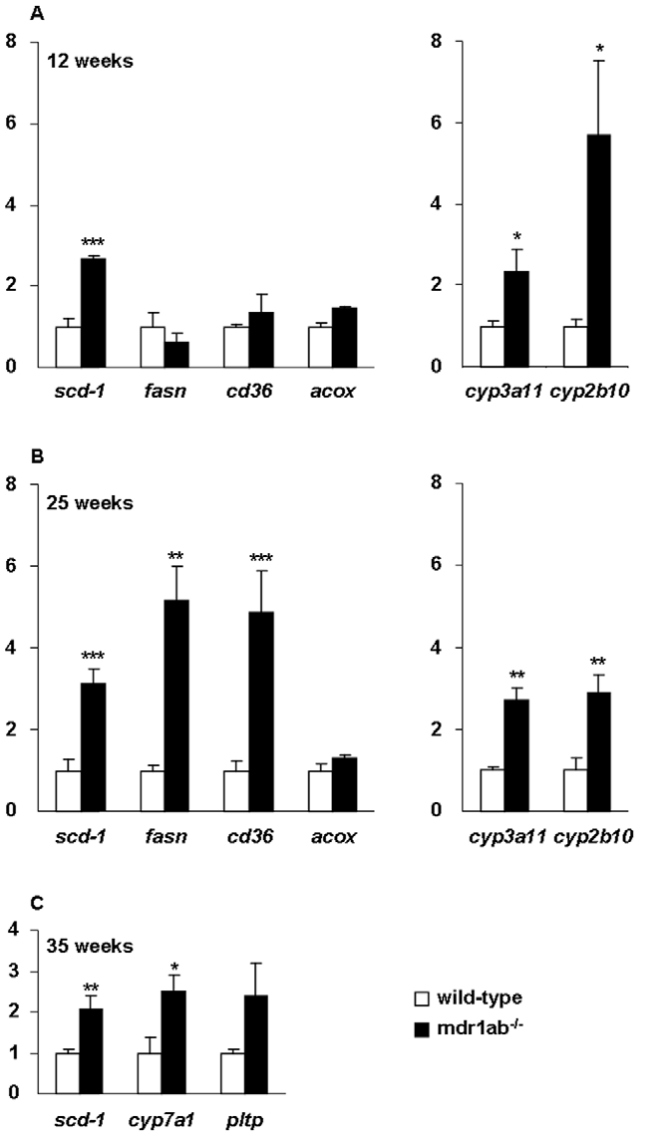
Analysis of lipids and xenobiotics target genes expression in the liver. Relative gene expressions of *scd-1*, *fasn*, *cd36*, *acox*, *cyp2B10* and *cyp3A11* were quantified in liver samples from 12-week old (Fig. 5A) and 25-week old (Fig. 5B) mice by real-time time PCR. Similarly, *scd-1, cyp7a1* and *pltp* expressions were quantified in 35-week old mice (Fig. 5C); wild-type (open bars) and mdr1ab-/- (solid bars). Values were normalized to TBP gene and expressed as arbitrary unit. Values shown are means ± s.e.m (n = 3). Differences are considered significant when *p*<0.05. *, *p*<0.05; **, *p*<0.01; ***, *p*<0.001.

To gain insight into the molecular mechanisms underlying the effects of Pgp deficiency on hepatic lipid composition, we have examined the liver expression of several representative genes involved in lipid homeostasis that are controlled by nuclear receptors activated by endogenous cholesterol and derivatives (liver X receptor, LXR) or by fatty acids (peroxisome proliferator-activated receptors, PPARα and PPARγ). We focused on genes coding for representative enzymes or transporters involved in fatty acid synthesis (stearoyl-coenzyme A desaturase, SCD-1, and fatty acid synthase, FASn), lipid uptake (cluster of Differentiation 36, CD36) and peroxisomal β-oxidation (fatty acyl-CoA oxidase, ACOX) at 12 and 25 weeks, ages corresponding for Pgp-deficient mice to normal weight and early overweight phenotype, respectively. At 12-week of age, only the expression level of *scd-1* was significantly increased in mdr1ab^-/-^ mice when compared with wild-type mice (2.5-fold, *p<*0.01, Fig. 5A) while at 25 weeks of age, simultaneous and significant increases of *fasn, scd-1* and *cd36* mRNA abundance occur in mdr1ab^-/-^ mice. At both ages, the expression of *acox* was unchanged by Pgp deficiency (Fig. 5B). In addition, in Pgp-deficient mice at 35 weeks the expression of two genes *cyp7a1* and *pltp* encoding cholesterol 7 alpha-hydroxylase (CYP7A), involved in bile acid formation and phospholipid transfer protein (PLTP), involved in cholesterol elimination were increased by 2.5-fold in the same way as *scd-1*.Given that Pgp is involved in xenobiotic transport, we also explored the expression of the cytochrome genes *cyp2b10* and *cyp3a11* which are modulated by xenosensors such as the constitutive active/androstane receptor (CAR, NR1I3), and the pregnane X receptor (PXR, SXR, NR1I2). At 12 weeks, both genes expressions were significantly increased with a massive increase of *cyp2b10* (6-fold, *p<*0.001, Fig. 5A). At 25 weeks both genes were 3-fold induced in Pgp-deficient mice when compared with wild-type mice (Fig. 5B).

## Discussion

The toxicological and pharmacological impacts of Pgp have been well established for many xenobiotic [4]–[7]. On the basis of its ability to transport a large range of lipids [8], [9], a role in lipid homeostasis has been evocated for Pgp but has never been clearly established *in vivo*.

Our study showed for the first time that the Pgp-deficient mice fed a standard chow diet developed an overweight phenotype which occurred earlier and was more pronounced in males than in females. Besides becoming overweight, a substantial increase of white abdominal, subcutaneous and visceral adipose tissue mass was observed with severe hyperinsulinemia which evolved to hyperglycemia.

Hypertrophy of visceral fat pads is considered as a predictive factor for lipid metabolic disorders linked to obesity and our data obtained in Pgp-deficient mice are consistent with the increased propensity of people who are overweight to develop insulin resistance *via* the oversupply of fatty acids to tissues. In addition, liver steatosis appeared clearly in overweight 25-week old Pgp-deficient mice as evidenced by the massive hepatic triglyceride accumulation, the increases in cholesterol and biliary acid in bile, and of ALP and ALT activities in plasma.

When Pgp-deficient mice were fed HFD, deregulation of metabolism occurred early. Plasma glucose and plasma and hepatic triglycerides were increased at 15 weeks when compared with matched wild-type mice fed HFD, revealing that Pgp deficiency leads to a sensitisation to high-fat diet.

Obesity is multifactorial and overeating is one of the obvious causes. Indeed, hyperphagia has been observed in several monogenic disruption obese mouse models (*ob* [22], *db* [23], *or agouti* [24]). Because the lack of Pgp did not notably influence feeding behaviour, we exclude overfeeding as the cause of the obesity in our model.

Obesity occurs in many situations where genes involved in lipid homeostasis are disrupted. The liver steatosis observed here evokes up regulation of *de novo* lipid synthesis in the liver. Indeed, *Fasn* and *scd-1* expressions were increased in the livers of Pgp-deficient mice and by their pivotal role in d*e novo* fatty acids synthesis they could be major contributors of hepatosteatosis. Overexpression of *scd-1* was observed in 12-week old Pgp null mice and appeared as an early event in the onset of lipid disorder. In accordance with our results, mice lacking SCD-1 are lean with significantly reduced triglyceride storage in the liver [25] and obesity is related to an increased expression of *scd-1* [15], [26]. Similarly, the overexpression of the well-known ubiquitous scavenger receptor CD36 in the livers of obese Pgp-deficient mice, may contribute to the onset of obesity by favouring lipid accumulation in tissues [27], [28]. At the same time, the *Acox* gene coding for a rate-limiting enzyme in β-oxidation of fatty acids, was not modified. This suggests that fat catabolism remains stable in our model.

These data are all the more interesting in that obesity occurred, as a consequence of Pgp deficiency, in a FVB genetic background which is known to be resistant to diet-induced obesity. Indeed, body weight and energy expenditure [15] or hallmark obesity genes, such as scd-1 [29], were unchanged in normal FVB mice fed high-fat diet, which is in contrast to C57BL/6 in which HFD rapidly induces an overweight condition and massive overexpression of *scd-1* [15]. In the same vein, obese transgenic C56BL/6*ob/ob* mice had massive increased expression of *scd-1* while only a moderate increase was observed in FVB*ob/ob* [26].

It is obvious that the gene expression profile described above led to unbalanced lipid homeostasis, contributed to hepatosteatosis and to the obesity phenotype in the Pgp-deficient mice. Given that *cd36*, *scd-1 and Fasn* are overexpressed in our model and that their expressions are regulated by the nuclear receptors PPARγ for *cd36* [30] and LXR for *scd-1 and Fasn* [31], respectively, we suggest that the turn-over of activators of these nuclear receptors, i.e., fatty acid-derived ligands or cholesterol, might be affected in Pgp deficiency.

The accumulation of triglycerides in the liver was concomitant with the strong increase in concentrations of bile acids and cholesterol measured in bile of 35-week old Pgp-deficient mice. Together with the increase of *cyp7a1* expression observed in Pgp-deficient liver, our data are in accordance with the role of the enzyme CYP7A1 in bile acid synthesis. In the same vein, given the role of PLTP in HDL remodelling, the increased expression of *pltp* was consistent with the increase of HDL in plasma of Pgp-deficient mice and attests of abnormal clearance of cholesterol. C*yp7a1* and *pltp* are direct targets of LXR [32], [33] and our results demonstrate that the cholesterol metabolic pathway is altered in Pgp deficiency. In support with our data, another study performed in mdr1ab^-/-^ mice fed HFD showed the increased levels of LXR protein and PLTP activity [34].

Nevertheless, the main role of Pgp is to extrude a broad range of chemicals and mice lacking Pgp accumulate significantly higher plasma and tissue concentrations of Pgp substrates such as glucocorticoids and xenochemicals [7] and certainly food components. We showed that in Pgp-deficient animals, the expression of *cyp2b10* and *cyp3a11* were increased, consistent with likely increases in the availability of ligands of the hepatic xenosensors PXR and CAR [35], [36]. The list of candidate stimuli that are transported by Pgp and that can influence P-450s, through CAR and PXR activation, is long and includes exogenous regulators of dietary origin, such as pesticides [37], flavonoids [38] and phytooestrogens [39], whose presence depends on the housing conditions. Indeed in previous works, *P-450* genes were shown to be differentially modulated in Pgp-deficient mice, depending on the controlled animal housing conditions and the origin of the standard chow [40].

The activation of CAR or PXR by xenobiotics may be contributing to obesity in Pgp-deficient mice, especially since the role of xenosensors has been expanded to the regulation of lipid metabolism and energy expenditure. Indeed, CAR has been shown to impact on serum triglyceride levels [41] and PXR increases hepatic deposit of triglycerides [42]. In addition, they both increase *de novo* hepatic lipogenesis by enhancing the expression thyroid hormone-responsive 14 protein [43]. Whether PXR and CAR activation, as a consequence of Pgp deficiency, is a direct cause of obesity and steatosis by disrupting genes involved in lipid homeostasis or whether gene induction by xenobiotics and by lipids are two independent pathways, remains to be determined.

Interestingly, in favour of a role for Pgp in lipid metabolism in humans, single nucleotide polymorphisms on the Pgp coding gene *ABCB1* have been associated with lipid metabolism disorders in several independent studies. In hypercholesterolemic patients, the haplotype G2677T and C3435T on *ABCB1* is associated with higher levels of total and LDL cholesterolemia [18]. Similarly, in hypercholesterolemic women the *ABCB1* allele G2677T/A was associated with higher levels of total and LDL cholesterol than those with the more frequent allele G [17]. While in STANISLAS cohort, G2677T/A polymorphism was associated with a decrease in plasma total cholesterol but this was in healthy subjects [16]. Moreover, among 95 polymorphisms of 67 candidate genes, explored in a Japanese cohort including 4252 individuals, only a Pgp gene polymorphism was clearly and significantly associated with obesity. The body mass index was greater for individuals with the Pgp gene variant 2677A/T allele than for those with the GG genotype [19]. Although such an allele is not associated with deficient Pgp function as we would have expected in accordance with our results, this study reinforces our finding of a strong link existing between Pgp and lipid homeostasis.

In humans, there is no genotype described so far associated with a full lack of Pgp function. Nevertheless, SNPs in the Pgp gene, including synonymous SNPs, have been associated with low Pgp function [44], [45]. In addition, therapy is sometimes based on using drugs which are strong inhibitors of Pgp. As an example, HIV patients chronically treated with antiretroviral protease inhibitors frequently elicit a metabolic syndrome including hyperlipidemia, lipodystrophy, and insulin resistance [46]. Although several mechanisms have been proposed to explain such drug-induced deregulations and because protease inhibitors are strong Pgp inhibitors, we cannot rule out that Pgp inhibition may contribute, to some extent, to these disorders.

Thus, our results are relevant in the context of people having low Pgp activity-associated polymorphisms and being exposed simultaneously to drugs (often used in combination), food contaminants and natural food components that can be Pgp inhibitors [47]. With such combinations, substantial inhibition of the transporter activity may occur and favour the onset of lipid metabolism disorders. Interestingly, Pgp polymorphism associated with a complete lack of Pgp function has previously been described in several canine strains [48], [49] but the link between obesity and Pgp deficiency has never been investigated so far in dogs.

Our data indicate that Pgp-deficient mice develop excess weight, metabolic disorders, hepatic steatosis that clearly characterise obesity. The metabolic disruption appeared earlier and was more severe in Pgp-deficient mice fed a high-fat diet. Overall, Pgp deficiency reverses the resistance to obesity phenotype which characterizes FVB mice. This is the first time that a clear obese phenotype has been described in Pgp-deficient mice since they have been generated [5]. Given the key role of Pgp in controlling the entrance of a broad range of compounds into the body, we thus propose that subsequent to Pgp inhibition, lipids or any active compounds may be absorbed at a higher rate and modulate target gene expression, affecting metabolic pathways that favour the occurrence of an obesity syndrome. These data reinforce the findings that some polymorphisms in the Pgp gene could be a predisposing factor and may be a relevant marker for obesity. Indeed, any decrease in Pgp function, due to simultaneous occurrence of polymorphism and chemical inhibition, may favour obesity. This can be even worse in occidental societies, where people are facing a dramatic excess in lipid dietary intake, which if combined with Pgp inhibition, may accelerate the onset of lipid metabolic disorders. Deciphering the mechanisms by which Pgp deficiency is involved in the development of obesity in mice may help reveal novel physiological functions for Pgp and elucidate factors associated with the onset of obesity.
